# Identification of a defense response gene involved in signaling pathways against PVA and PVY in potato

**DOI:** 10.1080/21645698.2020.1823776

**Published:** 2020-10-07

**Authors:** Zhila Osmani, Mohammad Sadegh Sabet, Kenji S. Nakahara, Ali Mokhtassi-Bidgoli, Khabat Vahabi, Ahmad Moieni, Masoud Shams-Bakhsh

**Affiliations:** a Department of Plant Genetics and Breeding, Faculty of Agriculture, Tarbiat Modares University, Tehran, Iran; b Research Faculty of Agriculture, Hokkaido University, SapporoJapan; c Department of Agronomy, Faculty of Agriculture, Tarbiat Modares University, Tehran, Iran; d Department of Cell and Metabolic Biology, Leibniz Institute of Plant Biochemistry, Friedrich-Schiller- University, Jena, Germany; e Department of Plant Pathology, Faculty of Agriculture, Tarbiat Modares University, Tehran, Iran

**Keywords:** G-proteins, stress response, transgenic potato plants, virus resistance

## Abstract

Potato is the most important non-grain food crop in the world. Viruses, particularly potato virus Y (PVY) and potato virus A (PVA), are among the major agricultural pathogens causing severe reduction in potato yield and quality worldwide. Virus infection induces host factors to interfere with its infection cycle. Evaluation of these factors facilitates the development of intrinsic resistance to plant viruses. In this study, a small G-protein as one of the critical signaling factors was evaluated in plant response to PVY and PVA to enhance resistance. For this purpose, the gene expression dataset of G-proteins in potato plant under five biotic (viruses, bacteria, fungi, nematodes, and insects) and four abiotic (cold, heat, salinity, and drought) stress conditions were collected from gene expression databases. We reduced the number of the selected G-proteins to a single protein, *StSAR1A*, which is possibly involved in virus inhibition. *StSAR1A* overexpressed transgenic plants were created via the Agrobacterium-mediated method. Real-time PCR and Enzyme-linked immunosorbent assay tests of transgenic plants mechanically inoculated with PVY and PVA indicated that the overexpression of *StSAR1A* gene enhanced resistance to both viruses. The virus-infected transgenic plants exhibited a greater stem length, a larger leaf size, a higher fresh/dry weight, and a greater node number than those of the wild-type plants. The maximal photochemical efficiency of photosystem II, stomatal conductivity, and net photosynthetic rate in the virus-infected transgenic plants were also obviously higher than those of the control. The present study may help to understand aspects of resistance against viruses.

## Introduction

Potato (*Solanum tuberosum* L.), as one of the most important agricultural crops in the world (FAOSTAT, http://faostat.fao.org), is central to global food security and ranks third after rice and wheat.^1^ Crop growth and yield can be drastically reduced by virus diseases. The loss in potato yield caused by viruses can reach up to 90% depending on the type of the virus.^2^ The main cause of seed degeneration was also found to be viruses, particularly potato virus Y (PVY) and potato virus A (PVA) of the genus *Potyvirus*.^3^ In plants, virus infection induces dramatic morphological and physiological changes such as decreased plant vegetative performance, decreased photosynthesis, and increased respiration. These adverse effects of viral infection on host plants result in inferior performance such as decreased host biomass and crop yield loss.^4,5^

Host resistance is one of the best strategies to control virus infection in plant species.^6–8^ One of the considerable challenges of potato breeders is to interpret the resistance mechanisms against virus and develop virus-resistant potato cultivars. Hence, knowledge about how plants regulate their innate immune response and how it can be manipulated by pathogens is necessary in plant science to identify and define natural plant response to stresses.^9–11^ Viruses must be capable of exploiting infected cell processes to replicate, translocate from one cell to another via plasmodesmata, and move systemically in the entire plant using the plant vascular system.^11,12^ Owing to the very limited viral genome size, RNA viruses encode only a small number of essential proteins. Therefore, it is crucial for the virus to employ host factors to promote the infection cycle efficiently.^11,13^

Defense signal transduction pathways involve various genes, which are critical for plant immune responses against diverse pathogens. Although several natural resistance genes against members of the genus *Potyvirus* have been reported in the recent decade in numerous plant species, few of them have been identified.^14^ Resistance (R) genes, which generally encode nucleotide binding and leucine-rich repeat (NB-LRR) proteins, deliberate a strong resistance called a hypersensitive response (HR). This resistance involves specific recognition of the pathogen effector, i.e. race specificity (gene-for-gene resistance).^15^ Therefore, resistance is ineffective in protecting the plants when new pathogen strains appear after a few years of use.^16^ Due to the high frequency of emerging resistance-breaking pathogens against R-genes, the focus now is on using signaling and defense-related genes for breeding.

To prepare an array of effective tools against viruses, plants have also evolved many other mechanisms, including production of biochemical compounds before or after the invasion of the pathogen into the plant tissues. Artificial induction of defense signaling and related genes in plants like potato has been shown to confer resistance to a broad-spectrum of pathogens using transgenes such as transcription factors (WRKY, ERF, TGA, MYB), kinases (MAPK kinases, CDPKs), negative regulators (RIN4, SNI1, SON1), and positive regulators (EDS1, PAD4, SGT1).^17–19^ Unlike breeding by introducing of R genes, the resistance acquired by manipulating downstream signaling and defense-related genes has been documented that can be durable and effective.^17,20–22^ This is true also for the members of signal-transducing G-proteins.

Guanosine triphosphate (GTP)-binding proteins (G-proteins) are essential components participating in signal transduction pathways derived from various stresses, including plant defense signaling.^23,24^ Upon binding with GTP, G-proteins are activated by the hydrolysis of the bound GTP to GDP.^25^ As many G-proteins are stress-responsive, active G-proteins bind with downstream targets to affect and initiate signaling events upon stresses.^24–26^ Three broad categories of signal-transducing G-proteins, including heterotrimeric G-proteins, small G-proteins (small GTPases), and other ‘unconventional’ G-proteins, have been identified in plants.^24,27^ The small GTPase superfamily is divided into four main subfamilies in plant eukaryotic cells: ADP-ribosylation factor 1 (ARF1)/secretion-associated RAS super family 1 (SAR1), Ras-related proteins in brain (RAB), Rho-related protein from plants (ROP), and RAs-related nuclear protein (RAN).^28–30^

Plants recognize the pathogen elicitors and produce a diverse array of primary and secondary signals in response to biotic and abiotic stresses to activate various plant protector and defense genes. Subsequently, the defense genes prevent pathogen invasion by producing glutathione S-transferases, proteinase inhibitors, peroxidases, PR proteins, and hydrolytic enzymes (downstream responses).^31^ Previous studies, regarding the plant defense response to pathogens, have shown that modification or overexpression of a single signaling pathway gene regulating a large number of defense-responsive genes confer resistance to a broad spectrum of pathogens.^18,19^ There are many examples of successful engineered plants by transformation with different constructs to overexpress trans- and endogenous genes in transgenic plants. Overexpression of signaling and defense-related genes can lead to a constitutive expression resistance phenotype.^32^ Small G-proteins play a vital role in plant disease resistance and subsequent cellular responses to pathogens such as bacteria,^33–35^ fungi,^36^ and viruses.^37,38^ A number of G-proteins have already been introduced into different plants. Overexpression of *OsRAN2*, a small G-protein in transgenic rice and *Arabidopsis thaliana* plants, exhibited higher survival rates under cold stress than the wild type did.^39^ Transgenic tobacco plants overexpressing a small G-protein, RGPL, enhanced resistance to tobacco mosaic virus (TMV) infection.^40^ It was also observed that expression of constitutively active (CA) *OsRac1*, a small GTPase Rho in transgenic rice plants, resulted in production of reactive oxygen species (ROS) and cell death.^41^ In another research, heterologous expression of N resistance gene and *OsRac1* in tobacco transgenic plants showed a failure to induce antioxidant genes against TMV infection resulting in impaired ROS production.^42^ In potato, stable overexpression of *AtRop1* (DN-*AtRop1*) increased resistance to fungus *Phytophthora infestans* infection.^43^

Among small GTP-binding proteins, SAR1 is a unique family within the Ras superfamily. Endomembrane trafficking plays a key role in the maintenance of fundamental cellular functions (signal transduction, cellular homeostasis, etc.) and in response to environmental stresses. SAR1, as a molecular switch, controls the assembly of the coat protein complex II (COPII) directing vesicle budding from the endoplasmic reticulum (ER)^44^ and integrates coat assembly with the cargo selection process.^45–47^ The COPII machinery is involved in plant responses to biotic and abiotic stresses.^46^ The SAR1 GTPase is an essential factor of COPII vesicle coats involved in plant responses to various stresses.^45,46^ Although the SAR1 superfamily of small GTPases is a key regulatory GTPase, its functions in response to various stresses are not well understood.

Plant genetic engineering represents an applied approach to traditional breeding methods, since it can elevate resistance to diseases, while other desirable traits of the plant are maintained. Finding differentially expressed genes under virus infection is an initial step to search for the most important genes in plant antiviral defense response. In this regard, candidate genes are likely to be significantly activated in resistant cultivars. In this study, given the ability of G-proteins in the enhancement of stress resistance, G-proteins were collected in potato plants infected with PVY and PVA using meta-analysis of publicly available microarray datasets. In this screening, a gene, encoding a kind of small G-protein, which was originally named as *StSAR1A*-like protein (*StSAR1A*) was selected. The selected transgenic plants were tested to determine whether overexpressed *StSAR1A* enhanced resistance to viruses.

## Materials and methods

### Microarray data sources

The microarray sets of the G-proteins in potato plants under virus stresses were collected from the microarray gene expression databases of NCBI GEO (http://www.ncbi.nlm.nih.gov/geo/) under the accession numbers GSE18196, GSE12041, GSE46180, GSE10903, GSE10488, and GSE8213 as well as ArrayExpress (http://www.ebi.ac.uk/arrayexpress/) under accession numbers E-MEXP-2622, E-MEXP-290, and E-MEXP-289. To investigate the expression level of the *StSAR1A* gene in potato plants under various stresses, the data of four biotic; bacteria (GSE8221), fungi (GSE8258, GSE13341, and GSE8250), nematodes (GSE8220 and GSE8209), and insects (GSE8255), and four abiotic; cold (GSE8203 and GSE8205), heat (GSE8202 and GSE8205), salinity (GSE18053, GSE8158, and GSE8205), and drought (GSE10481, GSE8161, and GSE8243) stresses were collected. Finally, a total of 113 differentially expressed genes (G-proteins) were selected in the following stresses: bacteria (13), fungi (36), nematodes (36), insects (20), cold (36), heat (23), salt (13), and drought (27).

### Data filtering

**Figure 1.**
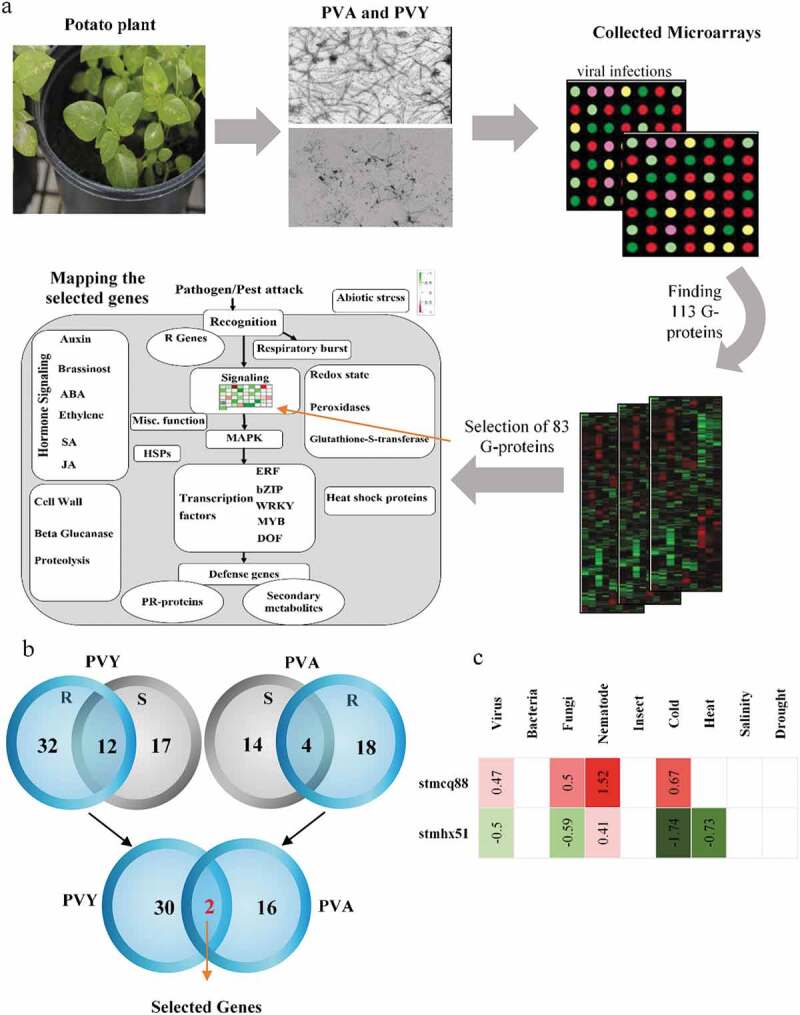
Overview of the systems biology-based approach for the candidate genes determination in potato plants (a) Microarray dataset from several infection experiments with PVY and PVA and MapMan analysis, (b) Venn diagrams to compare the host resistance and susceptibility data sets, and (c) Heatmap analysis of the selected genes in response to the biotic or abiotic stresses. The gene expression responses were calculated using the log2-transformed ratios from stress treatments (biotic or abiotic) and control experiments. Green and red colors donate down- and up-regulated genes, respectively and white color indicates no significant change between each stress and corresponding controls.

In our previous study, we showed for the first time, that a three-step filtering method identified key genes with higher confidence in details.^48^ In the present study, we reused the filtering method to identify the potential key genes by focusing on GTP binding genes. Briefly, the selected genes were filtered in a three-step process to detect the key genes that were effectively responsive to the PVY and PVA infection in potato plants. For this purpose, the differentially expressed genes (fold change ≥1.25 and *P* < .05) were obtained using Fisher’s combined probability test^49^ (Figure 1(a)). The genes exhibiting significant expression changes only in resistant plants were identified as follows. The pattern of gene expression after virus infection was separately compared in susceptible and resistant plants, as described previously.^48^ Using the Venn diagram, the number of shared genes was found between susceptible and resistant plants. A list of genes that were uniquely expressed in resistant plants was obtained by subtracting those expressed in susceptible plants. Since we aimed to select genes involved in resistance to both viruses, we reduced the number of the selected genes commonly expressed in both PVY and PVA infection experiments (Figure 1(b)).

### Cultivation and propagation of potato plants

Seed tubers of the potato (*S. tuberosum* L. cv. Desiree), which are susceptible to PVY and PVA, were planted in the greenhouse with natural light under a 16/8-h light/dark photoperiod and a temperature of 22°C. In this study, the plant *S. tuberosum* cv. Degima, which is resistant to PVY and PVA, was served as positive control plant. For propagation, plant nodes were cultivated on liquid MS^50^ containing salts (Wao, Osaka, Japan), vitamins, and sucrose (Table 1) in a growth chamber with 16/8 h light/dark photoperiods and under 22/18°C day/night temperatures.

**Table 1.** Media culture composition for transformation and regeneration of potato plants.

Ingredients	Concentration in culture medium			
	Propagation	Co-culture	Shoot regeneration	Rooting
MS salts	1 X	1 X	1 X	1 X
Nicotinic acid	0.5 mg L^−1^	1 mg L^−1^	1 mg L^−1^	1 mg L^−1^
Pyridoxine HCL	0.5 mg L^−1^	1 mg L^−1^	1 mg L^−1^	1 mg L^−1^
Thiamine HCL	0.1 mg L^−1^	10 mg L^−1^	10 mg L^−1^	10 mg L^−1^
Glycine	2 mg L^−1^	-	-	-
Myoinositol	100 mg L^−1^	100 mg L^−1^	100 mg L^−1^	100 mg L^−1^
Adenine sulfate	-	40 mg L^−1^	40 mg L^−1^	-
Glucose	-	20 g L^−1^	20 g L^−1^	-
Mannitol	-	20 g L^−1^	20 g L^−1^	-
Sucrose	30 g L^−1^	-	-	30 g L^−1^
Gibberellic acid	-	0.05 mg L^−1^	0.05 mg L^−1^	-
1-Naphthalene-acetic acid	-	0.02 mg L^−1^	0.02 mg L^−1^	0.05 mg L^−1^
Trans-zeatin-riboside	-	3 mg L^−1^	3 mg L^−1^	-
Agar-agar	-	8 g L^−1^	8 g L^−1^	-
Cefotaxime	-	-	300 mg L^−1^	300 mg L^−1^
Kanamycin	-	-	100 mg L^−1^	100 mg L^−1^

### Preparation of viruses for inoculation tests

In this study, PVY strain, infecting transgenic and control plants, was the ordinary strain of PVY (PVY^O^). The PVY isolate was from the laboratory collection at the University of Hokkaido, which was maintained as freeze-dried infected tobacco leaf material or as purified virus preparations at −20°C. The accession number of PVA used in this work was MAFF307028 from the NARO Genebank (https://www.gene.affrc.go.jp/databases-micro_search_detail.php?maff=307028).^51^ Infected leaves (100 mg) were ground well with 1 ml of 0.5 M phosphate buffer (0.1 M KH_2_PO_4_ and 0.1 M K_2_HPO_4_, pH 7.2). The crude supernatant was mechanically inoculated into third true leaves of three-weeks-old *Nicotiana benthamiana* plants. The plants were grown in a plant growth chamber with a 16/8 h light/dark photoperiod cycle at 25°C. Seven days after inoculation (dpi), upper non-inoculated leaves showing symptoms were used as an inoculum for mechanical inoculation into potato plants.

### Cloning of StSAR1A in a binary vector

The PCR was carried out using the gene-specific primers designed from 5ʹ untranslated region (UTR) and 3ʹ UTR (Table 2) to obtain the full-length coding sequence (CDS) of the *StSAR1A* gene (GenBank accession number KY196464) and the Max *Taq* DNA polymerase (mixture of *Taq* and *Pfu* in a 16:1 ratio; Vivantis Technologies, Malaysia) according to the manufacturer’s instructions. The purified PCR products were cloned into a pTZ57R/T cloning vector. The pTZ57R/T-*StSAR1A* vector was digested with the restriction enzyme (*Xho*I) to confirm the presence and length of the inserted fragment. After confirmation by sequencing, the correct pTZ57R/T-*StSAR1A* vector was cloned into the downstream of the cauliflower mosaic virus (CaMV) 35S promoter of pRI201-AN (10432 bp) as a plant binary vector (Takara Bio., Tokyo, Japan). *Agrobacterium tumefaciens* strain LBA4404 was then transformed with the constructed pRI201-AN-*StSAR1A* by the freeze-thaw method.^52^

**Table 2.** Primer sequences and amplified length for G-protein (*StSAR1A*), PVY (potato virus Y), PVA (potato virus A), *18S* (*18S* ribosomal RNA), 35S (CaMV 35S promoter) genes, *HSP* (heat shock protein terminator), and *Vir B6* (vir genes of Agrobacterium).

Amplicon	#	Sequence (5′→3′)	PCR product length (bp)
*StSAR1A*	F1	AGCTCGAGATCCCCATACCAATCG	783
	R1	GCTAAATAGATGCTTGATGCATACA	
	F2^a^	AGCATCAGCCGACTCAGTATCC	198
	R2^a^	GTGCGTCTAATTCTTTCTTTGACTCAG	
PVY	F^a^	ATACTCGGGCAACTCAATCACA	167
	R^a^	CCATCCATCATAACCCAAACTC	
PVA	F^a^	TTTCTATGAGATCACTGCAACCACT	116
	R^a^	TGACATTTCCGTCCAGTCCAA	
*18S*	F^a^	GGGGCATTCGTATTTCATAGTCAGAG	102
	R^a^	CGGTTCTTGATTAATGAAAACATCCT	
*StSAR1A*-T7	R^b^	AATTCTAATACGACTCACTATAGGGCTCGAGTAGATGCTTGATGCATACA	-
35S	F	TCTGCCGACAGTGGTCCCAA	-
*HSP*	R	ACACAAACTTAAGCACACAAGCTAG	-
*Vir B6*	F	CGTTTACGGCCATTCATACGATC	-
	R	GACTCCGAAGGCAGACCAGAG	

^a^Primers-sequence used for real-time PCR and ^b^primer-sequence used for Southern blot analysis; whereby T7 promoter-sequence is underlined.

### Genetic transformation of potato plants

Potato plants cv. Desiree was transformed with *A. tumefaciens*^53^ containing the pRI201-AN-*StSAR1A* binary vector. The internode pieces (4–6 mm) of potato tissues were used as explants for transformation. The explants were maintained on a co-culture medium (Table 1) with no antibiotics under dark conditions at 22°C for 3 d. For resistance screening, the internode samples were placed on the selected medium (shoot regeneration medium) (Table 1) under a light intensity of 30 µmol m^−2^ s^−1^ provided by daylight fluorescent tubes at 22–25°C for 2 months. The regenerated shoots were transplanted to a rooting medium (Table 1) with a light intensity of 20 µmol m^−2^ s^−1^ at 24°C for further growth and root development. The rooted transformants were transferred to plastic pots containing peat, perlite, and vermiculite (1:1:1 ratio) at 4–6 weeks of age under 16/8 h photoperiod at 25/22°C day/night temperature. The transformation efficiency index (%) presents the formula as follows:

[number of kanamycin-resistant regenerated explants]/[total number of explants cultured in selective regeneration medium with kanamycin and cefotaxime antibiotics] × 100.

In this study, the regeneration medium without antibiotic was used as a control medium. Kanamycin-resistant fully rooted plants were further tested for the presence of transgene using PCR.

### Molecular characterization of the transgenic plants

The kanamycin-resistant transformants were confirmed by PCR using the forward primer for the CaMV 35S promoter (35S-F) and the *StSAR1A* specific reverse primer (*StSAR1A*-R1) (Table 2) with the product 788 bp. Oligonucleotide primers (Table 2) were designed using the Primer Analysis software, Oligo Ver. 7.54 (Molecular Biology Insights; Wojciech and Piotr Rychlik, USA). The PCR-positive plants were further verified by Southern blot analysis. Total genomic DNA was separately isolated from the potato leaves of transgenic and non-transgenic plants, as the control, following the plant DNAzol reagent (Invitrogen, USA) according to the manufacturer’s instructions. The genomic DNA isolated from untransformed potato plants and pRI201-AN plasmids containing the transgene (*StSAR1A)* were used as negative and positive controls, respectively. The probe sequences were designed using the Oligo 7 Primer Analysis Software. To prepare hybridization probes, the pRI201-AN vector (pRI201-AN-*StSAR1A*) was used as a template in the PCR reaction. The T7 promoter-sequence was fused to the 5′-end of the *StSAR1A* gene-specific primer (*StSAR1A-*T7-R**, Table 2). The PCR was carried out using the gene-specific reverse primer with T7 (*StSAR1A*-T7-R**), the gene-specific forward (*StSAR1A*-F) primer (Table 2), and using the KOD FX Neo DNA polymerase (Toyobo, Osaka, Japan). The amplicons of the expected size (808 bp) were gel purified and quantified using a Nanodrop spectrophotometer (ND-1000, USA). Approximately 200 ng of PCR product was used for DIG-labeled RNA. Transcription was carried out using the T7 RNA polymerase, the DIG RNA Labeling Mix, and the purified PCR fragment (template DNA). The reaction was incubated at 37°C for 2 h. The genomic DNA from the transgenic and non-transgenic plants (20 μg) was digested with restriction endonuclease *Eco*RI, which was not cut within the T-DNA region. The digested DNA was fractionated in a 0.6% (w/v) agarose gel and blotted onto a nylon membrane (Roche, Germany), and then it was UV cross-linked. DNA blotting and hybridization were conducted as previously described.^54^ The hybridization signals were detected using the Detection Starter Kit I, according to the manufacturer’s instruction (Roche, Basel, Switzerland).

### Gene expression analysis and detection of viral infection

Virus-inoculated leaf tissues (1 g) of *N. benthamiana* were carefully ground in 1 ml phosphate buffer (0.5 M, pH 8.0) and used to inoculate transgenic and control plants with similar viral doses. One week later, the expression patterns of each targeted gene in the course of PVY and PVA inoculation in transgenic and control plants were monitored by real-time PCR. Conserved regions of viral coat protein (CP) were used to monitor each virus (PVY-F2* and R2* or PVA-F2* and R2*). The expression level of the *StSAR1A* gene was evaluated using gene-specific primers (*StSAR1A*-F2* and R2*) (Table 2). Each biological replicate was obtained via the average of three technical replicates. Two leaves from three potato plants were sampled and pooled to produce three biological replicates per treatment.

Total RNA was extracted using a Plant Total RNA Purification Kit (cat#TR02-150, Molecular Biology Tools). The reverse transcriptase enzyme (MmuLV, Fermentas) was used to synthesize cDNA by following the manufacturer’s instructions. Additionally, 1 μl of diluted cDNA (1:20 ratio for targeted genes and viruses, 1:100 ratios for internal control genes) was tested with iQ SYBR Green Supermix (Bio-Rad) in a final volume of 20 μl reaction under the following conditions: 95°C for 3 min (hot start), followed by 40 cycles of 95°C for 5 s, 60°C for 10 s, 72°C for 20 s (amplification) and 95°C for 30 s, 65°C for 30 s, 95°C for 30 s (melt). The *18S* ribosomal RNA (X67238) was used as an internal control to estimate the relative expression levels. To detect primer-dimer artifacts and ensure amplification specificity, primers specificity was assessed using a melting curve analysis. Primer efficiency was confirmed using serial dilutions of cDNA at a final concentration of 10^−1^, 10^−2^, 10^−3^, 10^−4^, 10^−5^. The 2^−ΔΔCt^ method was used to calculate the relative level of gene expression.^55,56^ Three independent experiments were performed to confirm the results. In this study, Wt (non-inoculated potato plants), Wt/Y and Wt/A (wild-type plants inoculated with PVY and PVA, respectively), R (Resistant plants), R/Y and R/A (resistant plants infected with PVY and PVA, respectively), and Wt/M, R/M, and T/M (wild-type, resistant, and transgenic plants mock-inoculated for both viruses) were grown under the same conditions and served as controls. The internode segments without *Agrobacterium* treatment were grown in unselective medium with no antibiotic as control plants.

### Enzyme-linked immunosorbent assay (ELISA)

The extent of viral infection in transgenic and non-transgenic plants was determined by double-antibody sandwich ELISA (DAS-ELISA)^57^ using polyclonal antibodies raised against PVY and PVA. Three weeks after inoculation with PVY and PVA, non-inoculated upper leaf samples from the same positions of potato plants were collected and examined for virus accumulation. Two leaves from three potato plants were sampled and pooled together to produce three biological replicates per treatment. Absorbance at 405 nm optical density was determined using an ELISA plate reader (ARVO MX 1420 MULTILABEL COUNTER). The triplicate absorbance readings for each sample were averaged and corrected by subtracting these values from the buffer blank triplicate readings. Virus-inoculated samples were those with average absorbance values greater than R (R = [mean ± 3 × standard deviation] of the negative controls).^58^ Blank, mock-inoculated, and resistant potato plants were used as negative controls, and the virus-inoculated leaves of potato wild-type plants were considered positive controls.

### Gas exchange and chlorophyll fluorescence measurements

The virus-infected transgenic potato and control plants were used to determine the possible effects of PVY and PVA on photosynthetic parameters. Analyses of the various photosynthetic parameters were simultaneously performed at the second and third leaf counting from the top of the plants. Two leaves of each plant and three to nine plants per treatment were used to perform measurements. The gas exchange parameters were taken with an LI-6400 portable photosynthesis system (LI-COR Inc., Lincoln, N.E., USA), and analyses of the major fluorescence parameters (*F_0_, F_m_*, and *F_v_*) of Chl were simultaneously conducted using a portable modulated fluorimeter (Plant Stress Meter, PSM Mark II, Biomonitor S.C.I AB, Umea, Sweden). Leaves were dark-adapted for 20 min before measuring of the fluorescence transient over 2 s and with an actinic stimulation at PPFD of 400 µmol photon m^−2^s^−1^. Maximal fluorescence (*F_m_’*) and minimal fluorescence (*F_0ʹ_*) were measured for light-adapted leaves (for 15 min). Variable fluorescence (*F_v_*) was calculated from the difference between *F_o_* and *F_m_* in dark-adapted conditions and variable fluorescence (*F_v_’*) from the difference between *Fo’* and *Fm’* in light-adapted conditions. The non-photochemical quenching of variable ChlF (*qN*) was determined in accordance with the equation *qN* = (*F_v_-F_v_’*)/*F_v_*.^59^

### Data analysis

Data filtering was accomplished with J-Express 2012 and Excel software programs to reduce the data. GoMapMan online tool (http://www.gomapman.org/) was used to match the gene functional annotations. Furthermore, the MapMan version 3.5.1R2 software (http://mapman.gabipd.org) was run to visualize the results and regulated pathways during virus–host interactions of potato gene annotations. Moreover, the Venn diagrams were created with Bioinformatics and Evolutionary Genomics online (http://bioinformatics.psb.ugent.be/webtools/Venn/).

All real-time PCR-MCA assays were performed using AriaMx Real-Time PCR System software. Prediction and analysis of transcription factor binding sites were conducted using the CiiiDER tool to predict potential transcription factor binding sites within endogenous *StSAR1A* and CaMV 35S promoter sequences. CiiiDER identifies those transcription factors that are significantly enriched in both promoters. Statistical analyses were conducted on the obtained dataset using the SAS software (version 8.2; SAS Institute, Cary, NC). Duncan’s multiple range test^60^ was used to identify significant differences (*P* < .05) in gene expression analysis among tested plants into homogenous subsamples of means that were not different from each other. One-way ANOVA was also used for statistical analyses of multiple groups and physiological parameters. In addition, the cluster heatmap (distance measure: Euclidean; clustering algorithm: Ward) was obtained through online web-based tools (https://www.metaboanalyst.ca/).

## Results

### Identification of candidate genes involved in resistance to PVY and PVA

The expression analysis of 113 G-proteins in potato plants under virus stress conditions indicated that the total number of differentially expressed genes under PVY and PVA treatments was estimated to be 83 genes. These genes were mapped using the MapMan software to organize and display differentially expressed genes in biotic stress pathways (Figure 1(a)). The number of genes regulated in potato resistant cultivars was 32 and 18 for PVY and PVA, respectively. To recognize the G-proteins involved in viral resistance response (PVY and PVA), the shared genes were also found between them in potato resistant cultivars. Based on the results, the number of common differentially expressed genes in resistant cultivars was estimated to be two G-proteins, namely *stmcq88* and *stmhx51* (Figure 1(b)). Unlike *stmhx51*, the *stmcq88* gene produced no antagonistic response to virus stresses and other biotic and abiotic stresses in this study (Figure 1(c)). Therefore, *stmcq88*, a G-protein (referred to as *StSAR1A* gene in this study), was selected to evaluate the effect of *StSAR1A* overexpression on PVY and PVA resistance.

### Genetic transformation and molecular characterization of transgenic plants

To investigate the effect of *StSAR1A* overexpression on resistance to PVY and PVA, we generated the overexpressed transgenic potato plants (referred to as *StSAR1A*-OE in this study). Node cultivation of potato plants was propagated on the liquid MS medium. All untransformed internode pieces were regenerated in the control medium without antibiotics, while no regeneration occurred in the presence of kanamycin but without cefotaxime. The transformation efficiency was approximately 33%. Finally, 15 independent transformants were obtained for the *StSAR1A* gene after regenerating shoots and rooting transformed plantlets. Among them, ten regenerated plants were proven to have the *StSAR1A* gene by PCR. These transgenic potato plants were also found to be negative for the presence of the Agrobacterium confirmed using *Vir B6* primers.

Southern blot using the Digoxigenin-labeled full-length cDNA sequence of *StSAR1A* as a probe confirmed that the *StSAR1A* gene was integrated into the potato genome (Fig. S1). Independent integration pattern of transgenes into the different transgenic potato genome caused a various size of the transgene on the blot. The number of integrated points into the transgenic potato genome ranged from one to five as compared to the negative control (non-transgenic potato plant). As Fig. S1 shows, two copies and one copy of the transgene *StSAR1A* were presented in lanes T_1_ and T_2_ (red arrows), respectively in the genome of potato. Many factors influence transgene expression, including the transgene copy number. Integration of multiple copies of the transgene into the plant genome has mostly been considered a disadvantage and strongly related to inactivation/silencing of the transgene.^61,62^ Previous studies have also suggested for screening of single-copy transgene insertion events in potato plants.^63,64^ In the present study, approximately 50% of the insertions appeared to be single copy. Therefore, five transgenic events with one transgene copy number were chosen for subsequent analysis. The pRI20-AN vector with target genes (*StSAR1A*) was examined as a positive control.

### Assay of overexpression of StSAR1A transgene in transgenic plants

The expression of the *StSAR1A* transgene in non-transgenic and screened transgenic plants was analyzed using the real-time PCR. Analysis of melting curves for primer speciﬁcity showed good specificity with a single sharp peak. The results also demonstrated good primer efficiency for the internal control gene (95.1%), the *StSAR1A* gene (106.5%), and the coat protein gene of PVY (94.0%) and PVA (91.1%) (a slope – 4.4, – 2.9, – 4.5, and – 4.6, respectively).

**Figure 2.**
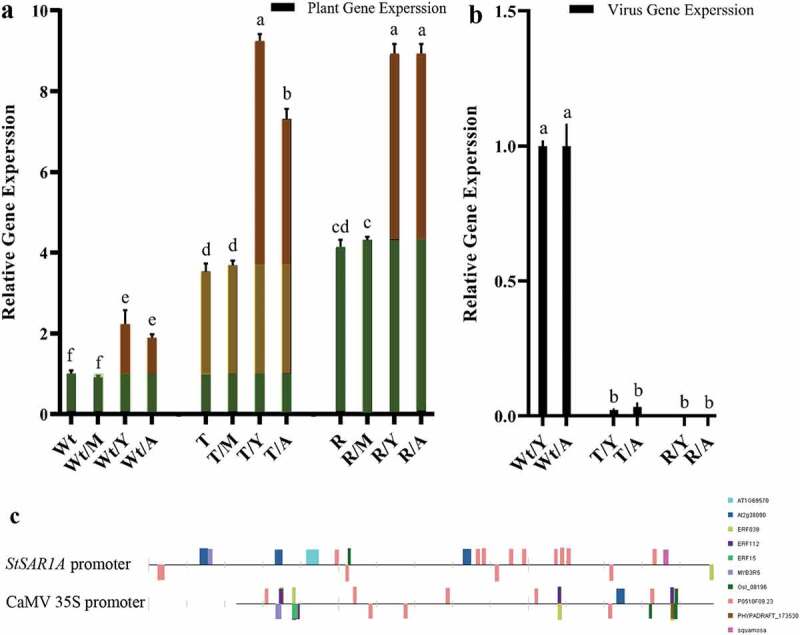
The relative expression levels of the potato *StSAR1A* gene and the virus coat protein gene in the transgenic and non-transgenic potato plants using real-time PCR. (a) relative expression level of *StSAR1A* gene at the same developmental stages. Green box, basal levels of the endogenous *StSAR1A*; Red box, induction of the endogenous *StSAR1A* under virus stress conditions (PVY and PVA), and Yellow box, the level of transgene *StSAR1A* under the control of the CaMV 35S promoter in the transgenic plants. (b) the expression level of potato virus Y (PVY) and potato virus A (PVA) coat protein gene in transgenic overexpressed plants compared to those in control ones. (c) Prediction and analysis of transcription factor binding sites using the CiiiDER tool to predict the potential transcription factor binding sites within endogenous *StSAR1A* and CaMV 35S promoter sequences. Colored boxes represent transcription factor binding sites, which are introduced with the same color on the right. Wt; wild-type potato plants cv. Desiree, R; resistant potato plants cv. Degima, T; transgenic potato plants cv. Desiree. In each case, M denotes mock-inoculated plants and Y and A denote plants infected with PVY and PVA, respectively. Mock-inoculated plants (Wt/M and T/M) and resistant potato plants (R/Y and R/A) were used as negative controls. Wild-type plants (Wt/Y and Wt/A) were used as positive control. The values represent the mean (± SE) from three biological replicates with three replicates each. Values plotted are averages of the fold changes for the five events in each treatment group shown in ± SE. The error bars indicate standard error of the average values of five events. The relative expression levels of the potato *StSAR1A* gene and virus CP genes were calculated by the 2^–ΔΔCt^ method with the *18S* rRNA gene as an internal control. In each case, bars having the common letter indicate non-significant differences, according to the Duncan’s Multiple Range test at the *P* < .05 level compared to Wt.

No statistical differences were detected between the non-inoculated (Wt, R, and T) and mock-inoculated (Wt/M, R/M, and T/M, respectively) plants in the expression level of the potato endogenous *StSAR1A* (Figure 2(a)). A low basal level of the endogenous *StSAR1A* was shown in potato Wt plants under non-stress conditions (Figure 2(a), green box), which can be used by plants in the routine activity. The ability of the wild-type plants to induce the endogenous *StSAR1A* upon viral infection was determined by comparing the gene expression levels of the endogenous *StSAR1A* in Wt/Y and Wt/A to those of Wt plants. Similar to Wt plants, there were extremely low expression levels of the endogenous *StSAR1A* gene in Wt/Y and Wt/A (2.2‐ and 1.9‐fold, respectively) (Figure 2(a)). The results indicated that the endogenous *StSAR1A* in Wt/Y and Wt/A was slightly induced (2.2‐ and 1.9‐fold, respectively) upon viral infection (Figure 2(a), red boxes). The results in the present study showed that the expression levels of *StSAR1A* in T plants were not significantly different from those of R plants. Significant differences were detected between R/Y and R plants or R/A and R plants in the expression level of the endogenous *StSAR1A* gene (Figure 2(a), red boxes). The results also revealed that the expression level of *StSAR1A* was significantly higher in T/Y and T/A plants than in Wt/Y and Wt/A plants (4.2‐ and 3.9‐fold, respectively). To assess the increased expression levels of the transgene *StSAR1A* under the control of the CaMV 35S promoter, the gene expression levels in T and Wt were compared to each other. The results showed that the fold induction of the transgene *StSAR1A* under the CaMV 35S promoter was significantly higher in T than in Wt (2.53-fold) (Figure 2(a), yellow boxes). With the aim to evaluate the induction of the endogenous *StSAR1A* in transgenic potato plants after virus infection, the gene expression levels of the endogenous *StSAR1A* in T/Y and T/A were compared to those in T. Interestingly, the results indicated that the fold induction of the endogenous *StSAR1A* was significantly higher in T/Y and T/A than in T (1.62‐ and 1.07‐fold, respectively (Figure 2(a), red boxes). Therefore, the induction of the endogenous *StSAR1A* after virus infection was higher in T/Y and T/A than in Wt/Y and Wt/A, respectively (3.15‐ and 2.88‐fold, respectively) (Figure 2(a), red boxes). Analysis of transcription factor binding sites was also conducted to predict the potential transcription factor binding sites within endogenous *StSAR1A* and CaMV 35S promoter sequences. The results revealed that these promoter sequences shared ten different transcription factors (Figure 2(c)).

### Assay of virus resistance in transgenic plants

To evaluate the effect of *StSAR1A* overexpression on resistance to PVY and PVA, we compared responses of transgenic plants against each virus and compared them to those of wild-type plants using the real-time PCR analysis. Overexpression of *StSAR1A* led to a reduction in the expression level of PVY and PVA CP genes up to 0.22- and 0.34-fold in T/Y and T/A plants relative to those in Wt/Y and Wt/A, respectively (Figure 2(b)). These results suggested an inverse relationship between the expression level of the *StSAR1A* gene and the viral CP gene. Hence, the difference in the expression level of the viral CP gene in viral-infected transgenic plants was not statistically significant compared to resistant plants. The results of this comparison showed that resistance of T/Y and T/A had the least differences compared to those of R/Y and R/A plants, respectively.

**Figure 3.**
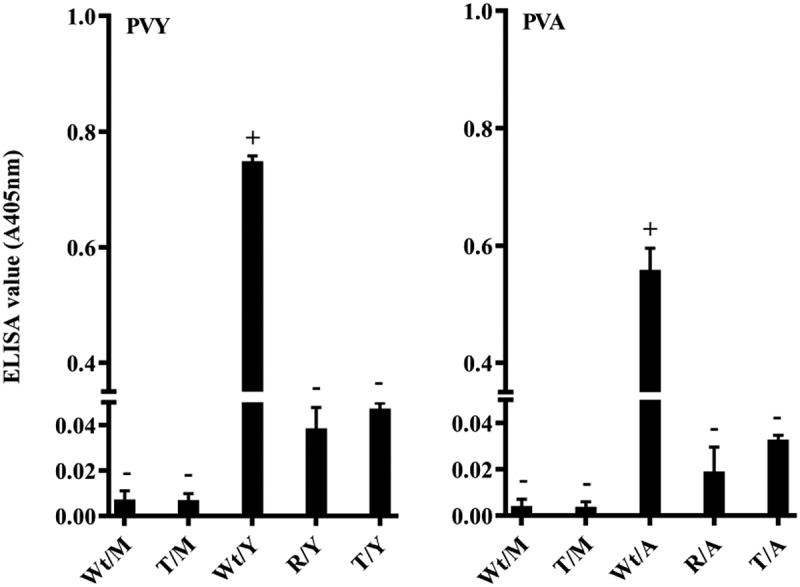
Investigation of the potato virus A (PVA) and potato virus Y (PVY) resistance of transgenic potato plants overexpressing *StSAR1A* gene and control plants. Accumulation of PVA and PVY coat proteins determined by enzyme‐linked immunosorbent assay (ELISA) of leaf samples from upper inoculated transgenic and non‐inoculated control plants at 21 dpi. Wt/M and T/M) Wild-type and transgenic mock-inoculated potato plants, R/Y and R/A) the resistant potato plants inoculated with virus Y and A as negative control in ELISA test. Wt/Y and Wt/A) the wild-type potato plants inoculated with each virus were used as positive control in ELISA test. The values represent the mean (± SE) from three biological replicates with three replicates each. The error bars indicate standard error of the average values of three biological replicates.

Virus CP accumulation in the leaves of studied plants was also investigated by ELISA at 21 d.p.i (Figure 3). The ELISA results were consistent with those of the real-time PCR. These virus accumulations were undetectable in mock-inoculated (Wt/M and T/M) and resistant (R/Y and R/A) potato plants as negative controls. In addition, PVY and PVA CPs accumulated at high levels in wild-type potato (positive control) plants. As a result, upper non-inoculated leaves of T/Y and T/A reduced the amount of virus CP compared to those in Wt/Y and Wt/A, respectively (Figure 3).

### Assessment of morphological traits

**Figure 4.**
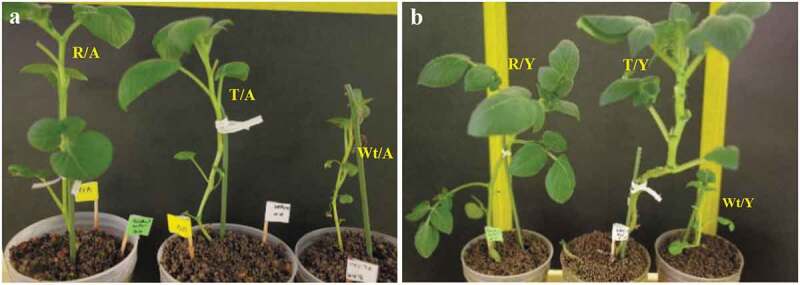
Comparison of the potato virus Y (PVY) and the potato virus A (PVA) symptoms of the *StSAR1A*-OE potato plants (T/Y and T/A), the resistant potato plants inoculated with each virus (R/Y and R/A), and wild-type plants inoculated with each virus (Wt/Y and Wt/A) three weeks after inoculation. (a) and (b) show PVA and PVY inoculated plants, respectively. Wild-type and resistant inoculated plants were used as a control.

We monitored plant growth under controlled conditions to assess phenotypic differences between transgenic and control potato plants. Although Wt/Y and Wt/A plants displayed chlorosis and less growth, PVY showed the most severe symptoms compared to PVA in the wild-type control (Figure 4). No apparent morphological differences existed between the non-inoculated (Wt, R, and T) and mock-inoculated (Wt/M, R/M, and T/M, respectively) plants in morphological traits (Table S1). Morphological responses of potato plants to virus stresses showed a significant decline in plant growth-related traits of Wt/Y and Wt/A compared to healthy control plants (Wt) (Table S1). The T/Y and T/A plants also showed phenotypic differences in plant growth traits compared to Wt/Y and Wt/A plants, which exhibited a quicker growth rate, a greater stem length and a larger diameter, a larger leaf size, a higher fresh/dry weight, and a greater node number. Similar data were obtained in R/Y and R/A plants than in Wt/Y and Wt/A plants, respectively (Table S1 and Figure 4). Moreover, T/Y and T/A plants showed a shorter internode length than Wt/Y and Wt/A plants did, respectively (Table S1 and Figure 4). No deleterious consequences of genetic transformation (e.g. pleiotropic or insertional effects) were observed on the plant morphology or growth in T/Y and T/A plants (Figure 4).

### Assessment of photosynthesis and gas exchange variables

Mock-inoculated plants (Wt/M, R/M, and T/M, respectively) did not show any changes compared to non-inoculated plants (Wt, R, and T) in terms of gas-exchange and photosynthesis parameters (Table S1). PVY and PVA infection of wild-type plants (Wt/Y and Wt/A) had severe impacts on photosynthetic responses compared to healthy control plants (Wt) (Table S1). Photosynthetic responses of *StSAR1A*-OE plants showed that various traits and parameters related to gas-exchange and photosynthesis responses were enhanced in both T/Y and T/A plants compared to Wt/Y and Wt/A plants (Table S1). The results revealed that transgenic inoculated plants exhibited reduced susceptibility to both PVY and PVA compared to wild-type plants. Like R/Y and R/A plants, T/Y and T/A plants, compared to Wt/Y and Wt/A plants, showed higher rates of *Fv*/*Fm*, stomatal conductivity and transpiration, net photosynthetic rate, intercellular CO_2_ concentration, and leaf temperature. On the contrary, for Wt/Y and Wt/A plants the opposite effect (i.e., higher *F’v*/*F’m* and qN) was observed compared to other plants.

### Physiological and morphological evaluation of the non-transgenic and transgenic potato responses to viruses by heatmap

**Figure 5.**
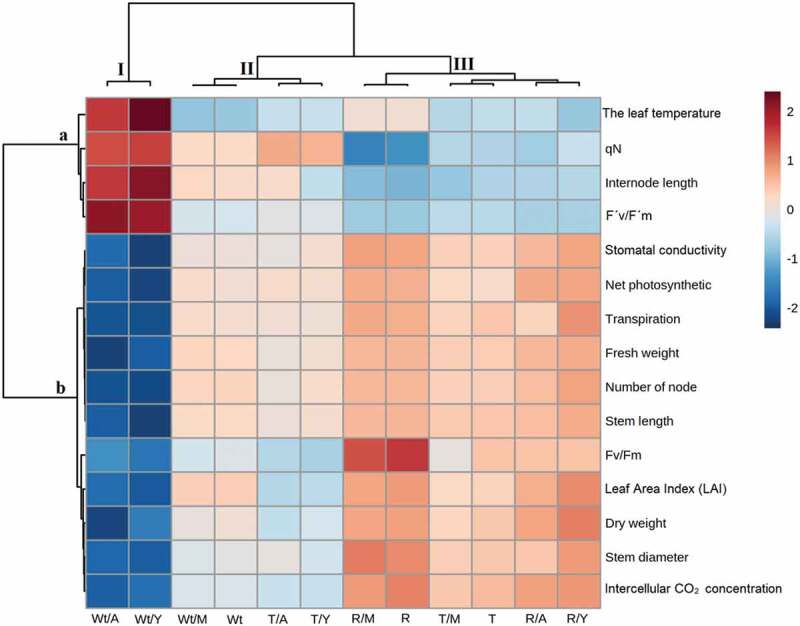
Overall assessments of morphological and physiological responses in non-transgenic and transgenic potato plants at the same developmental stages 21 d post inoculation with PVY and PVA. Wt; wild-type potato plants cv. Desiree, R; resistant potato plants cv. Degima, T; transgenic potato plants cv. Desiree. In each case, M denotes mock-inoculated plants and Y and A denote the plants infected with PVY and PVA, respectively. Mock-inoculated plants (Wt/M and T/M) and resistant potato plants (R/Y and R/A) were used as negative controls. Wild-type plants (Wt/Y and Wt/A) were used as positive control. Scale: from brightest blue equals most decreased to brightest red equals most increased.

The effect of virus infection on plant physiological and morphological traits and the overexpression of *StSAR1A* on the overall growth and development of transgenic plants were evaluated via heatmap hierarchical clustering (Figure 5). Clustering analysis (top) showed three major groups: Group I includes Wt/Y and Wt/A, Group II includes Wt, Wt/M, T/Y, and T/A, while Group III includes R, R/M, R/Y, R/A, T, and T/M. The clustering analysis (left) of different parameters showed two main groups where the Group a represented four studied physiological and morphological parameters (Tleaf, F´v/F´m, internode length, and qN), while Group b represented the other physiological and morphological parameters (Figure 5).

## Discussion

Plants have the ability to integrate divergent signaling pathways to allow appropriate defense responses against various stresses.^65,66^ Therefore, overexpression of a single gene can have a negative (i.e., susceptibility) or positive effect (i.e., tolerance) on the plant response to other stresses. In this study, the response evaluation of two selected genes; *stmhx51* and *stmcq88* to different biotic and abiotic stresses showed that unlike the virus stresses, the fungi, cold, and heat stresses caused a significant decrease in the expression level of the *stmhx51* gene (synergistic action with virus stresses) in potato resistant cultivars against every stress. There is an exception in potato resistant cultivar against nematode attack, which induced a significant increase of transcript levels of *stmhx51* (antagonistic action with virus stresses). Regarding *stmcq88*, the results of the heatmap support the idea that there is a synergistic action between virus and different stresses. Since the virus response gene, *stmcq88*, is also regulated by fungi, nematodes and cold stresses, previous studies have suggested that many components of the regulatory networks were antagonistically involved in responses to biotic and abiotic stresses’ function. These components can induce more or less susceptibility to a certain type of stress.^66,67^ In this study, analysis of gene expression in response to different biotic and abiotic stresses revealed that the *stmcq88* gene might not act antagonistically against the virus infection stresses. Moreover, the overexpression of the *stmcq88* gene may also increase resistance to fungi, nematodes, and cold stresses at the same time.

The R/Y and R/A have shown high ability to induce the expression of endogenous *StSAR1A* to Wt/Y and Wt/A, respectively. This comparison confirmed the inability of Wt plants to adequately induce the internal gene after virus infection. The results in the present study demonstrated that similar to R/Y and R/A plants, the expression levels of *StSAR1A* in T/Y and T/A plants were significantly different from those of Wt/Y and Wt/A, respectively. Therefore, overexpression of the *StSAR1A* transgene not only increases the capability of transgenic potato plants in adequate induction of endogenous *StSAR1A*, but also confers the virus resistance to PVY and PVA in T/Y and T/A plants, respectively. A positive interaction between a strong and weak promoter has been documented wherein the weakened promoter and powerful and contiguous promoter (such as CaMV 35S) synergistically affect each other. It has also been shown that the activity of CaMV 35S and weak promoters positively would influence each other when the weak and strong contagious promoters share the same transcription factors.^68^ The promoter analysis of CaMV 35S and *StSAR1A* gene showed that they shared some different transcription factors (Figure 2(c)). These observations suggest that the endogenous *StSAR1A* promoter might have been positively upregulated by the CaMV 35S promoter. Therefore, dramatic induction of overexpression *StSAR1A* in the transgenic potato after infection with virus (T/Y and T/A) relative to the infected wild type (Wt/Y and Wt/A, respectively) can be outcome of the positive interaction between a strong transgene promoter (CaMV 35S) and a weak endogenous promoter sharing the same transcription factors.

According to our results, the overexpression of the *StSAR1A* gene, as G-protein, significantly enhanced resistance to both PVY and PVA, suggesting the involvement of *StSAR1A* in antiviral defense potato plants. Considerable evidence indicates that plant endomembrane trafficking system is closely associated with stress-related signaling pathways to meet the requirements of rapid changes in cellular processes and to ensure the correct localization of stress‐related cargo molecules.^69,70^ However, the detailed molecular mechanisms are unknown. The function of SAR1A is critical for the assembly and organization of COPII, an important complex for protein transport from ER to Golgi.^45–47^ The COPII machinery is associated with plant responses to biotic and abiotic stresses.^46^ Our data suggest that SAR1A expression likely enhances virus stress resistance with the involvement of the COPII network, but this requires further study. Small G-proteins have also been proven to function in cell signaling events,^26^ especially in the regulation of plant immunity, as a key molecular switch for signaling pathways and their related functions in plants.^24,71^ Previous studies demonstrated the role of G-protein in defense against pathogens. Overexpression of the *OsGAP1* gene, encoding a small G-protein in rice genome, in transgenic *A. thaliana* and rice led to enhanced resistance to the bacterial pathogens in both monocots and dicots.^33^ Additionally, overexpression of *StRab*, a small GTP-binding protein gene from potato, significantly reduced the lesion area of the inoculated transgenic potato plants with mixture races of *P. infestans*.^36^ Small G-proteins were also found to be involved in the interaction between plant resistance genes and pathogen avirulence genes, activation of the other G-proteins to initiate defense responses, accumulation of ROS, and expression of PR proteins.^72,73^ Furthermore, the physical association of GTPase-activating proteins (GAPs) with NB-LRR resistance protein Rx has been shown to regulate against plant viruses.^37,38^ However, the way a small G-protein *StSAR1A* is involved in antiviral defense is an interesting question for further research. The overexpression result of *StSAR1A* in transgenic plants was the reduced rate of viral accumulation. Previous findings demonstrated that the small G-protein was involved in activating immune responses by activating the ROS signaling.^42,74^ Therefore, the small G-protein *StSAR1A* may enhance antiviral resistance possibly by regulating the ROS production in plants that could be explored in future studies. One of the earliest plant’s defense responses during an incompatible host–pathogen interaction is the generation of hydrogen peroxide (H_2_O_2_) and other ROS.^75^ H_2_O_2_ and ROS are important to prevent pathogen accumulation through antimicrobial activities.^42^

Modification or overexpression of a single signaling pathway gene may cause growth repression and/or yield loss due to constitutive overexpression of a large number of genes at a time.^19^ Virus infection causes a significant decline in plant growth-related traits, leading to crop yield loss. In this study, it can be concluded that overexpression of *StSAR1A* gene promotes the plant vegetative growth than wild-type plants when infected with viruses. In the present study, the potato transgenic plants were slightly affected by virus infection and showed a greater plant height, a larger node number, and a shorter internode length than wild-type-infected plants. Similar trends were observed in these characters between transgenic plants and resistant cultivars under virus infection. This is in agreement with previous reports in which virus infection drastically reduced the plant growth of susceptible cultivars, and ultimately resulted in crop losses compared to resistant cultivars.^76,77^ However, economic injury to plants due to virus infection depends on some important factors such as virus strain, resistance of the infected cultivar, the plant age at the time of infection, and environmental conditions.^78^

As demonstrated on the hierarchical clustering and heat map in Figure 5, Wt/Y and Wt/A were affected by the virus more than others were, and they fell in the separated clade. In addition, transgenic plants under stress conditions (T/Y or T/A) tended to cluster with wild-type under non-stress conditions (Wt). Therefore, transgenic potato plants can be considered PVY and PVA resistance owing to their better performance under stress conditions for all the studied traits. Moreover, under non-stress conditions, transgenic plants (T) tended to cluster with resistant plants, suggesting that transgenic and resistant plants have more or less similar response to virus according to the measured traits in Table S1. This result also indicated that overexpression of *StSAR1A* had no discernible effect on the growth and development of transgenic plants. Therefore, these plants can be considered for use in future breeding programs.

The heatmap of variables suggests that the leaf temperature, qN, Internode length, and F´v/F´m have been more affected in Wt upon virus infection (Wt/Y and Wt/A). On the contrary, these traits have been less affected by virus infection in resistant and transgene plants (Figure 5). These traits have been demonstrated as important markers of the plant. The internode length, for example, was positively correlated with the plant performance,^79^ and longer internodes help the plant to increase light harvest.^80^ Potato performance is reduced by increasing the temperatures above 25°C.^81^ Higher leave temperatures are also known to reduce potato canopy photosynthesis.^82^ Furthermore, it has been shown that leave high temperature negatively affects the leaf area development in potato plants.^83^ The effective photochemical efficiency in light-adapted state (FV’/FM’) can be used to estimate the PSII operational efficiency.^84^ Moreover, FV’/FM’ were positively correlated with lower plant photosynthesis and total yield efficiency in potato crop.^85^ The results demonstrated in Figure 5 indicated that over-performance of T/Y and T/A plants could improve the leaf temperature, qN, internode length, and F´v/F´m factors relative to Wt/Y and Wt/A.

Virus-infected sensitive plants often exhibit severe morphological and physiological changes, with modifications in the structure and function of the chloroplast.^4,5,86^ Reduction in the photosynthetic rate in sensitive plants is commonly reduced by virus infection.^4,87,88^ It has also been found that reduced plant growth rates in virus-infected plants can be associated with impaired photosynthetic apparatus.^4,86,87^ This study revealed improvement in the photosynthetic rate, which may lead to increased growth and fitness in T/Y and T/A plant than in Wt/Y and Wt/A plants, respectively. In line with this result, it has been reported that overexpression of small G-proteins can regulate stress resistance, promote plant growth, and increase yield production.^89,90^ Our results suggest that the photosynthetic activity of infected plants was more affected in PVY than in PVA. Consistent observations were reported previously.^5^ The findings of this research are also consistent with previous studies revealing that overexpression of *AtSAR1A* did not affect the plant growth negatively.^91,92^

Identification and understanding of the host cell factors would increase the knowledge of molecular mechanisms underlying the manipulation by viruses and subsequently may facilitate the development of a defense strategy against viruses. Identification of defense-related genes induced against virus infection would be useful to breed crop plants for antivirus resistance. Overall, our results revealed that *StSAR1A* was involved in defense responses against PVY and PVA in potato plants. Furthermore, it was found that we could confer enhanced antiviral resistance on potato plants by overexpression of the *StSAR1A* gene. This study demonstrates that our systems biology approach can be successfully applied for selection genes to confer beneficial traits and enhance resistance against various abiotic and biotic stresses like antiviral resistance on potato plants.

## Conclusion

In this study, we presented a strategy to select effective candidate genes and to examine their efficiency in practice. Screening of a list of G-proteins using microarray meta-analysis based on the systems biology approach suggested one candidate gene (*StSAR1A*). Virus-inoculation tests with transgenic plants revealed that constitutive expression of *StSAR1A* enhanced the resistance of transgenic potato plants against PVY and PVA. The experimental data were in agreement with meta-analysis regarding the enrollment of *StSAR1A* in the virus resistance process. In addition, morphological investigations revealed that *StSAR1A*-OE plants promoted plant growth and photosynthetic responses and did not show any negative impacts on the growth of transgenic plants. Since the *StSAR1A* gene was significantly up-regulated in response to PVY and PVA infection in all microarray experiments, *StSAR1A* may be one of the key regulatory genes during various plant–virus interactions, including PVY and PVA infection. Furthermore, the virus-infected transgenic plants exhibited a promoted growth rate, indicating the functionality of *StSAR1A* in potato growth. Morphological and physiological assessments and expression analysis of *StSAR1A* genes together demonstrated that *StSAR1A* worth to be implemented in the breeding programs not only because of it simple genetic regulation but also because it reacts to the virus-like resistant lines. Moreover, not only similarity of morphological and physiological responses between the resistant plants and transgenic plants, but also results of the expression analysis of *StSAR1A* gene (Figure 2) suggest the same regulatory and subsequently physiological programing during confrontation with the viruses. This issue leads to higher plant performance relative to Wt plants, demonstrating that selection of *StSAR1A* based on data analysis is an effective strategy to increase plant performance against viruses. Hence, *StSAR1A* would be a proper candidate for breeding potato plants highly resistant to viruses, perhaps with additional beneficial traits. Additionally, it is speculated that *StSAR1A* is important to modulate plant resistance to fungi, nematodes, and cold stresses as demonstrated by the meta-analysis method. Considering the involvement of small G-proteins in diverse biotic and abiotic stress responses, it is worth examining whether *StSAR1A*-OE potato plants have enhanced resistance or tolerance to multiple biotic and abiotic stresses. Further investigation of the physical interaction of the *StSAR1A* gene with downstream stress-responsive and growth and developmental-related genes is also necessary at the protein level.

## Supplementary Material

Supplemental Material

Supplemental Material

## Abbreviations

PVY: Potato virus Y
PVA: Potato virus A
*F_m_*: The maximal fluorescence in the dark-adapted state
*F_m_’*: The maximal fluorescence in the light-adapted state
*Fv*: Variable fluorescence in the dark-adapted state
*Fv’*: Variable fluorescence in the light-adapted state
*F_v_/F_0_*: The ratio of efficiency of electron donation to photochemical efficiency of photosystem II (PSII)
*F_0_*: The fluorescence intensity at 50 μs
*q_N_*: The non-photochemical quenching of variable ChlF
Wt: Wild-type potato plants cv. Desiree
Wt/Y: Wild-type potato plants cv. Desiree inoculated with PVY
Wt/A: Wild-type potato plants cv. Desiree inoculated with PVA
T: Transgenic potato plants cv. Desiree
T/Y: Transgenic potato plants cv. Desiree inoculated with PVY
T/A: Transgenic potato plants cv. Desiree inoculated with PVA
R: Resistant potato plants cv. Degima
R/Y: Resistant potato plants cv. Degima inoculated with PVY
R/A: Resistant potato plants cv. Degima inoculated with PVA
Wt/M: Wild-type potato plants cv. Desiree mock-inoculated for both viruses
T/M: Transgenic potato plants cv. Desiree mock-inoculated for both viruses
R/M: Resistant potato plants cv. Degima mock-inoculated for both viruses

## Key Message

To explore additional genetic resources to confer antiviral resistance on potato, we developed a screening and evaluation system using microarray meta-analysis and transgenic plants and exemplified it with small G-proteins.
