# Supplementary material for: Identification of a defense response gene involved in signaling pathways against PVA and PVY in potato
Source: GM Crops Food. 2020 Oct 7;12(1):86–105. doi: 10.1080/21645698.2020.1823776 (PMC7553743; doi:10.1080/21645698.2020.1823776)
Supplement: Supplemental Material [file KGMC_A_1823776_SM8705.docx]

| Table S1. Values of gas exchange, chlorophyll fluorescence parameters and morphological traits in the resistant, transgenic, and Wilde-type plants inoculated with PVA (R/A, T/A, and Wt/A, respectively), PVY (R/Y, T/Y, and Wt/Y), buffer (R/M, T/M, and Wt/M), and without inoculation (R, T, and Wt). The number of the replicates in each plant for each treatment was shown as Rep. No. The variables were measured in 21-day-old plants on growth chamber. Values represent the mean (± SE) of the replicates per treatment. Values within the same row followed by the same letter (s) are not significantly different (Duncan's multiple range test, *P* < 0.05) | | | | | | | | | | | | |
| --- | --- | --- | --- | --- | --- | --- | --- | --- | --- | --- | --- | --- |
| R/A | R/Y | R/M | R | T/A | T/Y | T/M | T | Wt/A | Wt/Y | Wt/M | Wt | **Genotype** |
| 6 | 6 | 3 | 3 | ***** | ***** | 3 | 3 | 6 | 6 | 3 | 3 | **Rep. No.** |
| 72.7 ± 1.45  **b** | 79.0 ± 0.58 **a** | 74.0 ± 0.58 **b** | 74.0 ± 1.15 **b** | 60.6 ± 0.75 **e** | 64.6 ± 0.74 **d** | 68.3 ± 0.67 **c** | 69.0 ± 0.58 **c** | 11.0 ± 1.15 **f** | 9.0 ± 0.58 **f** | 67.3 ± 0.42 **cd** | 67.0 ± 0.58 **c** | **Number of node** |
| 140.3 ± 0.88 **b** | 144.3 ± 0.33 **a** | 141.7 ± 0.67 **ab** | 142.3 ± 1.45 **ab** | 129.2 ± 0.82 **e** | 131.8 ± 0.64 **d** | 138.0 ± 0.58 **c** | 139.0 ± 0.58 **bc** | 82.7 ± 1.45 **f** | 75.7 ± 1.2 **g** | 134.0 ± 0.37 **d** | 133.7 ± 0.67 **d** | **Stem length (cm)** |
| 1.77 ± 0.09 **def** | 1.80 ± 0.06 **de** | 1.50 ± 0.05 **ef** | 1.43 ± 0.09 **f** | 2.37 ± 0.05 **c** | 1.90 ± 0.08 **d** | 1.60 ± 0.06 **ef** | 1.77 ± 0.14 **def** | 3.53 ± 0.15 **b** | 3.97 ± 0.09 **a** | 2.40 ± 0.1 **c** | 2.43 ± 0.12 **c** | **Internode length (cm)** |
| 10.0 ± 0.58 **b** | 11.0 ± 0.57 **a** | 11.7 ± 0.33 **a** | 11.3 ± 0.88 **a** | 8.7 ± 0.08 **c** | 8.1 ± 0.08 **d** | 9.8 ± 0.09 **b** | 10.0 ± 0.15 **b** | 3.9 ± 0.06 **e** | 3.7 ± 0.12 **e** | 8.6 ± 0.16 **cd** | 8.3 ± 0.12 **cd** | **Stem diameter (mm)** |
| 147.2 ± 0.72 **b** | 151.4 ± 2.36 **a** | 147.0 ± 0.58 **b** | 147.9 ± 0.09 **b** | 127.7 ± 0.46 **g** | 131.2 ± 0.58 **f** | 139.3 ± 1.45 **cd** | 140.8 ± 1.48 **c** | 48.5 ± 1.44 **i** | 58.1 ± 1.34 **h** | 136.5 ± 0.61 **de** | 136.7 ± 0.88 **e** | **Fresh weight (g)** |
| 14.6 ± 0.18 **b** | 15.4 ± 0.41 **a** | 14.5 ± 0.12 **b** | 14.7 ± 0.22 **ab** | 11.1 ± 0.11 **g** | 11.6 ± 0.13 **f** | 13.1 ± 0.18 **cd** | 13.6 ± 0.34 **c** | 5.9 ± 0.1 **i** | 7.7 ± 0.39 **h** | 12.6 ± 0.21 **e** | 12.3 ± 0.88 **de** | **Dry weight (g)** |
| 4.1 ± 0.09 **abcd** | 4.3 ± 0.09 **a** | 4.2 ± 0.03 **abc** | 4.2 ± 0.15 **ab** | 2.9 ± 0.08 **e** | 3.0 ± 0.13 **e** | 3.7 ± 0.12 **d** | 3.7 ± 0.03 **cd** | 1.7 ± 0.09 **f** | 1.6 ± 0.18 **f** | 3.8 ± 0.02 **bcd** | 3.8 ± 0.03 **bcd** | **leaf area index (LAI)** |
| 0.19 ± 0.01 **e** | 0.19 ± 0.01 **e** | 0.18 ± 0.02 **e** | 0.17 ± 0.01 **e** | 0.28 ± 0.02 **b** | 0.27 ± 0.01 **bc** | 0.22 ± 0.01 **cde** | 0.21 ± 0.01 **de** | 0.65 ± 0.01 **a** | 0.64 ± 0.03 **a** | 0.25 ± 0.01 **bcd** | 0.26 ± 0.01 **bcd** | **F´v/F´m** |
| 0.90 ± 0.01 **b** | 0.90 ± 0.02 **b** | 0.93 ± 0.01 **a** | 0.937 ± 0.01 **a** | 0.867 ± 0.01 **d** | 0.865 ± 0.01 **d** | 0.883 ± 0.01 **bc** | 0.90 ± 0.01 **b** | 0.840 ± 0.01 **e** | 0.83 ± 0.01 **e** | 0.88 ± 0.03 **cd** | 0.877 ± 0.02 **c** | **Fv/Fm** |
| 0.65 ± 0.02 **e** | 0.67 ± 0.01 **de** | 0.59 ± 0.01 **f** | 0.60 ± 0.01 **f** | 0.75 ± 0.01 **b** | 0.74 ± 0.01 **bc** | 0.66 ± 0.01 **e** | 0.66 ± 0.01 **e** | 0.80 ± 0.01 **a** | 0.81 ± 0.02 **a** | 0.71 ± 0.01 **cd** | 0.71 ± 0.02 **d** | **qN** |
| 0.22 ± 0.01 **ab** | 0.24 ± 0.02 **a** | 0.24 ± 0.02 **a** | 0.24 ± 0.02 **a** | 0.19 ± 0.01 **c** | 0.20 ± 0.01 **bc** | 0.21 ± 0.01 **abc** | 0.21 ± 0.01 **abc** | 0.07 ± 0.01 **d** | 0.04 ± 0.02 **d** | 0.19 ± 0.02 **ab** | 0.19 ± 0.01 **ab** | **Stomatal conductivity**  **(mol m^-2^ s^-1^)** |
| 2.01 ± 0.25 **abc** | 2.42 ± 0.14 **a** | 2.28 ± 0.01 **ab** | 2.26 ± 0.01 **ab** | 1.87 ± 0.09 **c** | 1.85 ± 0.11 **c** | 2.01 ± 0.1 **abc** | 2.11 ± 0.08 **abc** | 0.44 ± 0.06 **d** | 0.41 ± 0.05 **d** | 1.90 ± 0.05 **bc** | 1.92 ± 0.1 **bc** | **Transpiration**  **(mol m^-2^ s^-1)^** |
| 8.25 ± 0.21 **ab** | 8.32 ± 0.5 **a** | 8.23 ± 0.22 **ab** | 8.17 ± 0.22 **ab** | 7.40 ± 0.19 **ab** | 7.31 ± 0.17 **b** | 7.46 ± 0.08 **ab** | 7.40 ± 0.13 **ab** | 3.82 ± 1.16 **c** | 3.43 ± 0.47 **c** | 7.23 ± 0.29 **ab** | 7.35 ± 0.23 **b** | **Net photosynthetic** |
| 231.5 ± 1.08 **ab** | 232.0 ± 1.15 **ab** | 231.8 ± 0.33 **ab** | 233.1 ± 0.43 **ab** | 219.0 ± 0.65 **e** | 218.4 ± 0.68 **e** | 227.6 ± 0.89 **c** | 228.6 ± 0.87 **bc** | 202.1 ± 1.55 **f** | 203.3 ± 2.65 **f** | 220.7 ± 0.45 **de** | 220.6 ± 0.9 **de** | **Intercellular CO2 concentration (µmol mol^-1^)** |
| 24.4 ± 0.12 **de** | 24.11 ± 0.04 **e** | 24.84 ± 0.04 **c** | 24.86 ± 0.02 **c** | 24.4 ± 0.08 **d** | 24.46 ± 0.1 **d** | 24.32 ± 0.03 **de** | 24.43 ± 0.09 **de** | 26.13 ± 0.1 **b** | 26.79 ± 0.19 **a** | 24.12 ± 0.04 **e** | 24.07 ± 0.07 **e** | **The leaf temperature** |
| * Five events with 6-9 replicates | | | | | | | | | | | | |
