# Supplementary material for: Identification of a defense response gene involved in signaling pathways against PVA and PVY in potato
Source: GM Crops Food. 2020 Oct 7;12(1):86–105. doi: 10.1080/21645698.2020.1823776 (PMC7553743; doi:10.1080/21645698.2020.1823776)
Supplement: Supplemental Material [file KGMC_A_1823776_SM8702.docx]

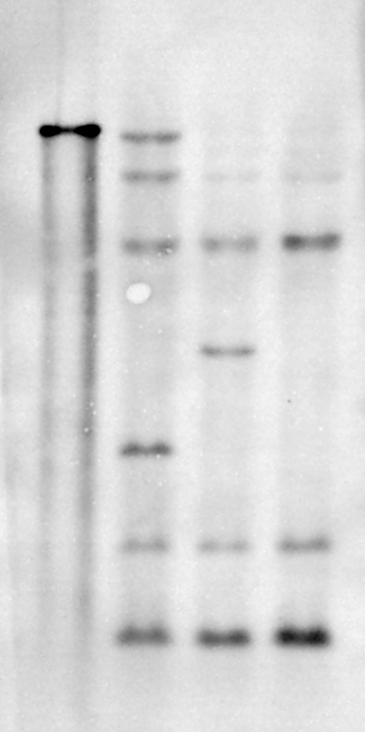


**C^+^ T_1_ T_2_ C^-^**

11220 bp

Figure S1. Southern blot analysis of transgenic potato plants overexpressing *StSAR1A* gene. T_1_ and T_2_) transgenic potato plants, C^-^) wild-type potato plant as a negative control, C^+^) the pRI201-AN-*StSAR1A* (11220 bp) binary vector containing the *StSAR1A* gene as a positive control; the full length coding sequence of *StSAR1A* labeled with Digoxigenin was used as the probe for Southern blot analysis, in which potato genomic DNA was digested with *Eco*RI. The red arrows indicate the copy number of integrated transgene in potato genome.
